# Effect of sodium–glucose co-transporter-2 inhibitors on the levels of serum asprosin in patients with newly diagnosed type 2 diabetes mellitus

**DOI:** 10.1186/s13098-021-00652-5

**Published:** 2021-03-25

**Authors:** Aijun Jiang, Zhanrong Feng, Lu Yuan, Ying Zhang, Qian Li, Yuqing She

**Affiliations:** 1 Department of Endocrinology, Nanjing First Hospital, Nanjing Medical University, 68 Changle Road, Qinhuai District, Nanjing, 21006 Jiangsu China; 2 Department of Endocrinology, Shuyang Hospital of Traditional Chinese Medicine, 28 Shanghai Middle Road, Shuyang, Suqian, Jiangsu China; 3 Department of Endocrinology, Nanjing Pukou Central Hospital, Nanjing, Jiangsu China

**Keywords:** Asprosin, Sodium-glucose co-transporter-2 inhibitors, Type 2 diabetes mellitus, Treatment

## Abstract

**Background:**

Asprosin, a novel adipokine that raises glucose levels and stimulates appetite, has been proved to be pathologically increased in populations predisposed to type 2 diabetes mellitus (T2DM), obesity, and cardiovascular diseases. The mechanisms of sodium-glucose co-transporter-2 (SGLT2) inhibitors for hypoglycemic effect and cardiovascular protection have not been fully clarified. Therefore, we conducted this study to assess change in the levels of serum asprosin after treatment with SGLT2 inhibitors in patients with newly diagnosed T2DM.

**Methods:**

This study was a randomized, double-blind, placebo-controlled trial. A total of 29 participants with newly diagnosed T2DM with body mass index (BMI) ≥ 23.0 kg/m^2^ and haemoglobin A1c (HbA1c) levels of 58–85 mmol/mol (7.5–10%) were randomized to SGLT2 inhibitors dapagliflozin 10 mg/d (n = 19) or placebo (n = 10) treatment for 24 weeks. We analyzed asprosin concentrations by an enzyme-linked immunosorbent assay. Besides, body weight, BMI, HbA1c, fasting plasma glucose (FPG), and lipid levels were measured at baseline and 24 weeks.

**Results:**

At 24 weeks, participants with SGLT2 inhibitors treatment exhibited lower levels of serum asprosin (22.87 vs 45.06 ng/ml in the placebo group; *P* < 0.001) after adjusting for baseline values. The levels of body weight, BMI, HbA1c, FPG, and triglyceride (TG) were decreased, while high density lipoprotein-cholesterol (HDL-C) was increased after SGLT2 inhibitors dapagliflozin treatment compared with placebo (*P* < 0.05 for all). Low density lipoprotein-cholesterol (LDL-C) and total cholesterol (TC) levels were unchanged in the SGLT2 inhibitors group and placebo group. No statistical correlation was found between the levels of serum asprosin and body weight, BMI, HbA1c, FPG, and lipid levels during the SGLT2 inhibitor dapagliflozin treatment.

**Conclusions:**

These findings indicated that SGLT2 inhibitors can lower serum asprosin levels and improve glucolipid and weight in patients with newly diagnosed T2DM, which may benefit the cardiovascular system.

*Trial registration* CTR20131268; Registered 20 March 2014 CTR20150102; Registered 03 March 2015. http://www.chinadrugtrials.org.cn/clinicaltrials.searchlistdetail.dhtml.

## Background

Type 2 diabetes mellitus (T2DM) is a serious chronic metabolic disease that puts a heavy burden on human health and social development worldwide [1]. Continuous hyperglycemia in T2DM patients induces harmful complications. Among them, Cardiovascular diseases are the major diabetic complications and increase mortality of patients with T2DM [2]. Additionally, T2DM patients are commonly accompanied by other cardiovascular disease risk factors, such as obesity and dyslipidemia [3]. Therefore, the focus on T2DM treatment has changed, showing as from "only hypoglycemia” to “comprehensive management of multiple risk factors”.

Asprosin, a novel adipokine, has been proved to be pathologically increased in patients with T2DM and obesity [4]. Asprosin is secreted by white adipose tissue, which acts on the liver, promotes hepatic glucose production, and raises glucose levels [5], and activates orexigenic agouti-related peptide (AgRP) neurons to stimulate appetite and accumulate adiposity and body weight [6]. Recently, the available evidence on the association of asprosin and cardiovascular diseases has also been discovered. Asprosin may be used as a biomarker to predict unstable angina pectoris and is shown that positively correlated with the degree of coronary stenosis [7]. Furthermore, its molecular mechanisms and function have been explored in other cardiovascular diseases, such as dilated cardiomyopathy [8] and myocardial infarction [9]. Thus, we hypothesize that asprosin is expected to be a therapeutic target for various metabolic diseases, particularly obesity, T2DM and cardiovascular diseases.

Sodium–glucose co-transporter-2 (SGLT2) inhibitors, a new type of hypoglycemic agent, selectively act on proximal renal tubules SGLT2 to inhibit glucose reabsorption and promote the excretion of glucose from the urine, which is independent of insulin resistance and islet function [10]. Several SGLT2 inhibitors are effective in controlling plasma glucose, including the improvement of hemoglobin (HbA1c) and fasting plasma glucose (FPG) [11–13]. Importantly, SGLT2 inhibitors have been confirmed their effects on weight loss [14] and cardiovascular protection [15]. These effects incompletely depend on the hypoglycemic effect, which has some unique mechanisms that may affect the levels of adipokines such as leptin and adiponectin involved in cardiovascular disease [16, 17]. Therefore, we evaluated the change in the levels of serum asprosin after SGLT2 inhibitors treatment in patients diagnosed newly T2DM, which may benefit to explain its mechanisms for cardiovascular system protection.

## Methods

### Participants

This study was a randomized, double-blind, placebo-controlled trial and was performed in the department of endocrinology, Nanjing First Hospital, Nanjing Medical University. Participants with newly diagnosed T2DM following the WHO diagnostic criteria (1999) [18] and drug-naïve type 2 diabetes, had HbA1c levels between 58 and 85 mmol/mol (7.5% –10%), body mass index (BMI) ≥ 23.0 kg/m^2^. The key exclusion criteria were severe hepatic and renal dysfunction, acute diabetic complication, suffered from acute or chronic pancreatitis at any time, have received or planned to undergo gastric bypass bariatric surgery or restrictive bariatric surgery during the study period, or long-term use of drugs that directly affect the motility of the gastrointestinal tract. We enrolled 29 participants, all of them completed the study. The protocol and informed consent document were approved by the ethics committee of Nanjing First Hospital, and all participants gave written informed consent before enrolling in the study.

### Study design and laboratory analyses

Eligible participants were assigned randomly to receive one of two treatments with dapagliflozin 10 mg/day or placebo, all are taken orally before breakfast every day. All participants have received instructions on a similar level of physical activity and the same nutritional value and equivalent energy intake of meals, and the original lipid-lowering and anti-hypertensive programs were maintained during the period.

At the beginning and end of 24 weeks of treatment, height, weight, and blood pressure of all participants were measured by a trained and certified nurse using standard protocols and techniques. After an overnight fast, fasting blood samples were acquired for the measurement of asprosin, FPG, HbA1c, and lipid profiles in all participants. Serum asprosin was determined by the enzyme-linked immunosorbent assay kit from Eiaab Science INC. Wuhan, China (Catalogue Numbers, E15190h). The kit had a sensitivity of 0.938 ng/mL, with a range between 1.563 ng/mL and 100 ng/mL. The intra-assay coefficient of variation is 6.6% and the inter-assay coefficient of variations 7.6%. The plasma glucose level was assessed by glucose oxidase method using an automatic biochemistry analyzer (HITACHI-7180, Tokyo, Japan), HbA1c was measured by high-pressure liquid chromatography (BIO-RADD-10 TM, California, USA), and lipid profiles were assessed by enzymatic colorimetric assay using an automatic biochemistry analyzer (HITACHI-7180, Tokyo, Japan). BMI was calculated by weight divided by the square of height (kg/m^2^). All these tests were done in the clinical laboratory of Nanjing First Hospital.

### Statistical analyses

All analyses were conducted using SPSS 26.0. all variables were tested for normal distribution by the Shapiro–Wilk test. Data conforming to the normal distribution were expressed as means ± standard deviation (SD). Paired t-test and independent-samples t-test were used to compare differences with groups. Data for non-normal distribution were expressed as the median and interquartile range (IQR), Mann–Whitney U test was used in the comparisons between groups. Pearson analysis in normal distribution variables and Spearman analysis in non-normal distribution variables were performed to identify the correlation between clinical and metabolic parameters. All comparisons were 2-sided at the 5% significance level. *P* value < 0.05 was considered to be statistically significant.

## Results

### Baseline characteristics

The study population consisted of 19 participants with T2DM in the dapagliflozin group and 10 in the placebo group. The demographic and baseline characteristics of study participants were balanced between two groups (Table 1).

**Table 1:** Baseline clinical characteristics of participants

	SGLT2 inhibitor (n = 19)	Placebo (n = 10)	Z/t	*P*
Age (years)	58.32 ± 8.01	59.3 ± 9.03	− 4.301	0.766
Gender (men/women)	9/11	2/8	/	/
Body weight (kg)	71.97 ± 8.31	66.20 ± 1.93	1.930	0.064
BMI (kg/m^2^)	26.60 ± 1.32	25.64 ± 1.41	1.809	0.082
SBP (mmHg)	123.20 ± 20.27	120.00 (120.00, 137.00)	− 0.786	0.432
DBP (mmHg)	78.16 ± 7.97	80.20 ± 9.31	− 0.612	0.541
BUN (mmol/L)	6.43 ± 1.30	5.82 ± 1.14	1.248	0.213
Cr (μ mol/L)	69.63 ± 1.30	67.10 ± 10.18	0.514	0.612
AST (U/L)	18.00 (15.00, 24.00)	19.00 (15.75, 29.25)	− 0.760	0.447
ALT (U/L)	17.00 (12.00, 30.00)	20.50 (15.25, 36.00)	− 0.599	0.599
ALP (U/L)	85.00 (4.00, 97.00)	80.50 ± 25.37	− 0.689	0.689
FPG (mmol/L)	9.12 ± 1.75	7.93 ± 0.84	2.018	0.054
Fasting C-pep (ng/mL)	0.52 ± 0.20	0.63 ± 0.20	− 1.507	0.143
2 h C-pep (ng/mL)	1.86 ± 0.87	2.11 ± 0.96	− 0.715	0.481
HbA1c (%)	8.10 (7.70, 8.90)	8.50 (8.05, 8.92)	− 0.965	0.334
TC (mmol/L)	4.88 ± 0.89	4.75 ± 2.18	0.238	0.814
TG (mmol/L)	1.71 (0.98, 3.40)	1.23 (1.04, 2.96)	− 0.964	0.335
LDL-C (mmol/L)	2.93 ± 0.83	2.342 ± 0.62	1.989	0.057
HDL-C (mmol/L)	1.28 ± 0.27	1.15 ± 0.31	1.170	0.252
asprosin (ng/mL)	36.88 (24.81, 69.04)	33.43 (19.64, 49.94)	− 0.620	0.535

*BMI* body mass index, *SBP* systolic blood pressure, *DBP* diastolic blood pressure, *BUN* blood urea nitrogen, *Cr* creatinine, *AST* aspartate transaminase, *ALT* alanine transaminase, *ALP* alkaline phosphatase, *FPG* fasting plasma glucose, *Fasting C-pep* fasting C-peptide, *2h C-pep* 2h C-peptide, *HbA1c* hemoglobinA1c, *TC* total cholesterol, *TG* triglyceride, *LDL-C* low density lipoprotein-cholesterol, *HDL-C* high density lipoprotein-cholesterol, *SGLT2* sodium–glucose co-transporter-2

### Effects of SGLT2 inhibitors on the levels of serum asprosin

**Fig.1:**
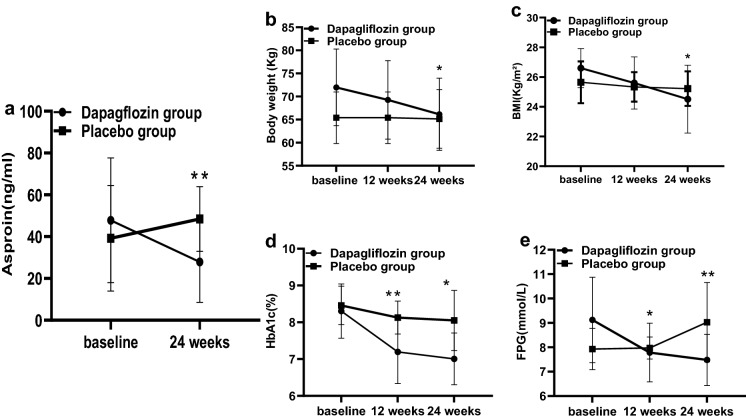
Change from baseline in asprosin, body weight, body mass index (BMI), glycated haemoglobin (HbA1c) and fasting plasma glucose (FPG) levels in the sodium–glucose transporter-2 (SGLT2) inhibitors and placebo group at baseline, 12 weeks and 24 weeks. *P < 0.01, **P < 0.001

At 24 weeks, the median levels of serum asprosin were significantly decreased in the dapagliflozin group from 36.88 to 22.87 ng/ml (*P* < 0.001). Furthermore, the median levels of serum asprosin with dapagliflozin treatment were significantly reduced to 22.87 ng/ml compared with 45.06 ng/ml with placebo treatment at 24 weeks (*P* < 0.001) (Fig. 1a).

### Effects of SGLT2 inhibitors on body weight and BMI

Modest but no significant changes were observed in body weight and BMI at 12 weeks in participants treated with dapagliflozin compared that of placebo (− 2.80 versus − 1.00 kg, and − 0.71 versus − 0.35 kg/m^2^; *P* > 0.05 for both). But these clear declines were significant at 24 weeks in the dapagliflozin group compared with the placebo group (− 5.83 versus − 1.06 kg and − 2.08 versus − 0.42 kg/m^2^; *P* < 0.05 for both) (Fig. 1b, c).

### Effects of SGLT2 inhibitors on glucose control

Decreased in HbA1c levels and FPG concentrations were both notable in the treatment group (Fig. 1d, e). At week 12 and 24, median changes from baseline in HbA1c levels were significant in the SGLT2 inhibitor group [− 12.13 mmol/mol (− 1.11%) and − 14.20 mmol/mol (− 1.30%), respectively] compared with the control group [− 3.60 mmol/mol (− 0.33%) and− 4.48 mmol/mol (− 0.41%); *P* < 0.001 and *P* < 0.05, respectively]. Compared with the placebo group, the treatment group exhibited a significantly greater reduction in FPG concentrations after 12 weeks and 24 weeks [(− 1.34 and − 1.64 mmol/L) versus (0.04 and 1.10 mmol/L), *P* = 0.005 and* P* < 0.001].

### Effects of SGLT2 inhibitors on lipids metabolism

Lipid metabolism measurements, including total cholesterol (TC), triglyceride (TG), high density lipoprotein-cholesterol (HDL-C), and low density lipoprotein-cholesterol (LDL-C) were assessed at baseline and 24 weeks. Decreased TG levels and increased HDL-C levels were detected with dapagliflozin treatment (− 0.34 mmol/L and 0.40 mmol/L, *P* < 0.05 for both). The differences in TC and LDL-C levels were both no statistically significant between the SGLT2 inhibitors group and the placebo group. (Table 2).

**Table 2:** Changes in lipids parameters at 24 weeks between groups with different treatment.

Parameters	SGLT2 inhibitor (n = 19)			Placebo (n = 10)			*P*-value b/w group at 24 weeks
	Baseline	24 weeks	*P*-value	Baseline	24 weeks	*P*-value	
TC (mmol/L)	4.88 ± 0.89	5.12 ± 0.85	0.061	4.75 ± 2.18	4.79 (3.77, 5.31)	0.785	0.094
TG (mmol/L)	1.71 (0.98, 3.40)	1.16 ± 0.56	0.020	1.23 (1.04, 2.96)	5.00 ± 1.78	0.878	0.501
LDL-C (mmol/L)	2.93 ± 0.83	3.15 ± 0.75	0.108	2.342 ± 0.62	1.94 ± 0.54	0.990	0.379
HDL-C (mmol/L)	1.28 ± 0.27	1.68 ± 0.39	< 0.001	1.15 ± 0.31	1.24 ± 0.25	0.173	0.012

*TC* total cholesterol, *TG* triglyceride, *LDL-C* low density lipoprotein-cholesterol, *HDL-C* high density lipoprotein-cholesterol, *SGLT2* sodium–glucose co-transporter-2

### Correlation analyses

Linear correlation analysis indicated that the lower levels of serum asprosin were not related to the change in body weight, BMI and glycolipid parameters.

## Discussion

There are several possible explanations for decreased serum asprosin and improved glucose levels with SGLT2 inhibitors treatment. Firstly, serum aspsorin is directly related to glucose metabolism and may be used as a potential therapeutic target for patients with T2DM. Zhang [4] found that the increased morbidity of T2DM is accompanied by higher asprosin levels and serum asprosin independently was associated with FPG and TG. Other clinical trials have analyzed that serum asprosin levels in healthy volunteers are significantly lower than those of patients with T2DM [19]. These results indicate that improved glucose levels are accompanied by deceased asprosin levels, which is consistent with results in our study. Secondly, Xu [20] reported the SGLT 2 inhibitors can increase the utilization of white adipose tissue, which is likely to explain the loss of body weight in patients treated with SGLT2 inhibitors in the current study and various randomized controlled trials [21, 22]. These affect SGLT2 inhibitors on enhancing adipose tissue expenditure, which could reduce the synthesis and release of asprosin that is secreted by white adipose tissue. Therefore, the lower levels of serum asprosin in the SGLT2 inhibitors group than in the placebo group after 24 weeks of treatment.

It is clear that mechanisms of asprosin are related to obesity, glucose, and lipid metabolism. Meanwhile, all of these are not good for cardiovascular system structure and function, particularly in patients with T2DM [23]. Actually, serum asprosin was found in the liver, brain tissue, and heart tissue in diabetic rats [24]. Besides animal experiments, a clinical study detected the direct relationship between serum asprosin and coronary artery stenosis in acute coronary syndrome people with unstable angina pectoris, their results showed that there was a significant positive correlation and asprosin may be the first biochemical marker for predicting the severity of unstable angina pectoris [7]. Thus, to some extent, decreased levels of serum asprosin are beneficial to the cardiovascular system in patients with T2DM.

SGLT2 inhibitors are distinguished from traditional hypoglycemic drugs because of their unique hypoglycemic effect and excellent cardiovascular protection [25, 26]. However, the cardiovascular protective mechanism of SGLT2 inhibitors is not completely understood [27]. Previous studies have found that SGLT2 inhibitors may benefit the cardiovascular system through reduced atrial natriuretic peptide and brain natriuretic peptide levels [28, 29]. Notwithstanding, researches have shown that adipokines involved in heart failure could be regulated by SGLT2 inhibitors [17, 30]. In the present study, the SGLT2 inhibitors reduced serum asprosin levels in patients with newly diagnosed T2DM, which may benefit to explain its mechanisms for cardiovascular system protection.

There are some limitations to the current study that should be taken into consideration. First, the study population was not large enough and the research time was relatively short. Second, more clinical parameters for explaining the mechanism of serum asprosin should be detected, such as waist-hip circumference and body fat analysis. Third, it is unknown whether better effects would be seen with different SGLT2 inhibitors dosage.

## Conclusions

In conclusion, we showed that SGLT2 inhibitors SGLT2 inhibitors can lower serum asprosin levels and improve glucolipid and weight in patients with newly diagnosed T2DM, which may benefit the cardiovascular system.

## Data Availability

All data generated and/or analyzed during this study are available from the corresponding authors upon reasonable request.

## Abbreviations

T2DM: Type 2 diabetes mellitus
SGLT2: Sodium–glucose co-transporter-2
BMI: Body mass index
HbA1c: Hemoglobin A1c
FPG: Fasting plasma glucose
TG: Triglyceride
HDL-C: Lipoprotein-cholesterol
LDL-C: Low density lipoprotein-cholesterol
TC: Total cholesterol
AgRP: Agouti-related peptide
SD: Means ± standard deviation
IQR: Interquartile range
